# Postnatal Management in Congenital Lower Urinary Tract Obstruction With and Without Prenatal Vesicoamniotic Shunt

**DOI:** 10.3389/fped.2021.635950

**Published:** 2021-04-14

**Authors:** Marietta Jank, Raimund Stein, Nina Younsi

**Affiliations:** ^1^Center for Pediatric, Adolescent and Reconstructive Urology, Medical Faculty Mannheim, University of Medical Center Mannheim, Heidelberg University, Heidelberg, Germany; ^2^Department of Pediatric Surgery, Medical Faculty Mannheim, University of Medical Center Mannheim, Heidelberg University, Heidelberg, Germany

**Keywords:** cLUTO, lower urinary tract obstruction, fetal therapy, vesicoamniotic shunt, surgical strategies

## Abstract

**Purpose:** Congenital lower urinary tract obstruction (cLUTO) includes a heterogeneous group of conditions caused by a functional or mechanical outlet obstruction. Early vesicoamniotic shunting (VAS) possibly reduces the burden of renal impairment. Postpartum, pediatric urologists are confronted with neonates who have a shunt in place and a potentially impassable urethra with a narrow caliber. Early management of these patients can be challenging. Here, we would like to share the approach we have developed over time.

**Materials and Methods:** We conducted a single-center retrospective analysis from 2016 to 2020 and included all patients diagnosed with cLUTO. Data focusing on time point and type of intervention was collected. Furthermore, patients with temporary diversion via a percutaneous VAS were selected for a more detailed review.

**Results:** In total, 71 cases of cLUTO were identified during the study period. Within this group, 31 neonates received postnatal management and surgical intervention in our center. VAS was performed in 55% of these cases (*N* = 17). The postnatal treatment varied between transurethral or suprapubic catheterization and early Blocksom vesicostomy. In five infants with VAS, the urinary drainage was secured through the existing VAS by inserting a gastric tube (*N* = 1) or a 4.8 Fr JJ-stent (*N* = 4). To our knowledge, this is the first report of a stent-in-stent scheme, which can remain indwelling until the definite treatment.

**Conclusion:** Having a secure urine drainage through a VAS allows the often premature infant to grow until definite surgery can be performed. This avoids placing a vesicostomy, which requires anesthesia.

## Introduction

Congenital lower urinary tract obstruction (cLUTO) is an obstructive uropathy that occurs in 3.3/10.000 births and predominantly affects male newborns (1). The most common underlying condition is “classical” posterior urethral valves (PUV), accounting for ~56–63% of the cases (1–4). Others include anterior urethral valves, obstructive ureterocele, as well as urethral hypoplasia, stenosis and agenesis. Due to the outlet obstruction, severe oligo- or anhydramnios can occur resulting in pulmonary hypoplasia and potentially neonatal death. Of those who survive, up to a third need renal replacement therapy (5–7).

The diagnosis is mainly based on antenatal ultrasound examination. Characteristic findings include bilateral hydronephrosis with megacystis +/- thick-walled bladder. Fetal megacystis refers to a bladder diameter of ≥7 mm in the first trimester. In the second trimester, it is defined as an abnormal distension of the thick-walled bladder failing to empty within 45 min of observation However, half the cases of megacystis show spontaneous resolution of the megacystis (8).

Due to improved antenatal, ultrasound-based diagnosis, early fetal intervention - predominantly vesicoamniotic shunting (VAS) – is possible starting from the 12–13^th^ week of gestation. VAS bypasses the obstruction, restoring amniotic fluid volume. As a result, it seems to improve the pulmonary as well as the renal function leading to decreased perinatal morbidity and mortality.

Assuming that fetal kidneys start producing urine at the 10th week of gestation and that nephrogenesis takes place in the first and second trimester, some authors argue that early VAS (up to the 14th week of gestation) would significantly improve kidney function. However, a detection of co-existing syndromes or other severe, life-threatening malformations is extremely difficult at this early stage of pregnancy.

In this situation, precise and sustainable medical counseling of prospective parents poses a serious challenge.

Nevertheless, postpartum pediatric urologists are confronted with a newborn having one or multiple artificial shunts in place and a urethra often lacking a sufficient caliber for cystoscopy within the first weeks of life.

Moreover, the percutaneous shunt is often an insecure form of urine drainage. Currently, the Harrison fetal bladder stent and the Somatex® intrauterine shunt are most frequently used for vesicoamniotic shunting. The Harrison stent is a 5 Fr flexible catheter with a curled tip on both sides, whereas the Somatex® consists of a wire mesh and self-deploying parasols at both ends. Recently published literature showed an intrauterine dislocation rate between 45% (Somatex® shunt) and 85% (Harrison stent) (9). Postnatally, the newborn's growth and steady movement increases the risk of shunt dislocation, overgrowth and granulation. In our opinion, relying on the vesicoamniotic shunt as the only diversion is unsafe. Therefore, a safe temporary solution until definite treatment is needed. Options include transurethral catheterization or early vesicostomy. When a VAS with adequate drainage is in place, percutaneous placement of a suprapubic catheter is usually not possible or very difficult. However, placing an external tube through the shunt to drain the urine like a suprapubic catheter may be a solution.

Objective of this study is to draw the readers' attention to the current research gap on the immediate surgical postnatal management of these patients and present possible solutions and approaches. Hence, we want to share our 4 years of experience in treating patients with cLUTO *with* and *without* VAS after birth. Based on our analysis and results, we aim to facilitate and attempt to standardize postnatal care of newborns with cLUTO.

## Materials and Methods

After institutional ethical approval (Ethical Approval No. 2020-873R), we performed a retrospective analysis of patients with cLUTO treated at our tertiary care center between 1^st^ of January 2016 and 30^th^ of September 2020.

**Figure 1:**
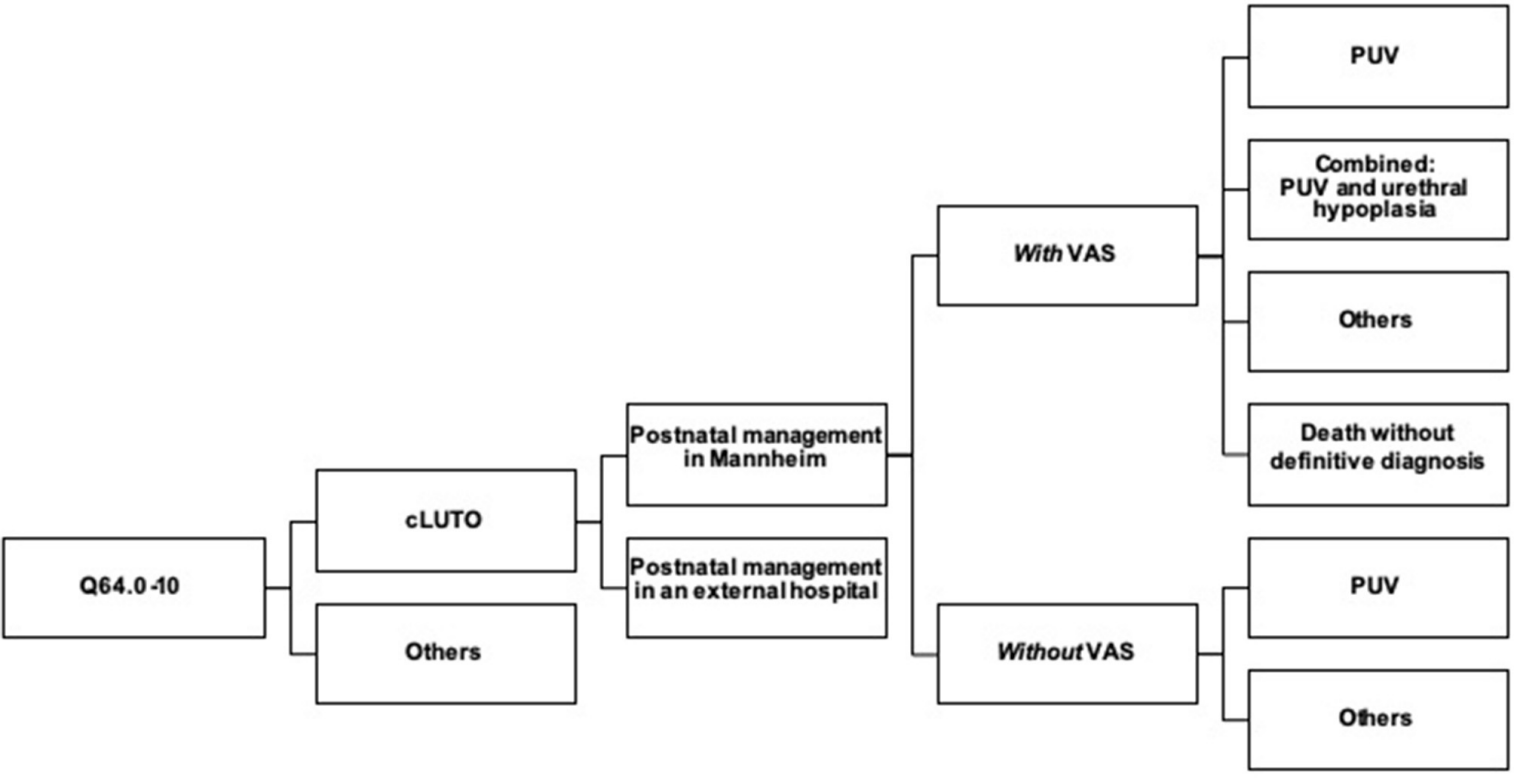
Flowchart describing the 31 cases of cLUTO treated in our center after birth. cLUTO: congenital urinary tract obstruction, PUV: posterior urethral valves, VAS: vesicoamniotic shunting.

For our initial data collection, we identified male and female patients with cLUTO aged 0–18 years using the ICD-10 code Q64.0-10 (congenital anomaly of the urinary system). The diagnoses meatal stenosis, exstrophy-epispadias complex and urachal anomalies were excluded and patients with a subvesical obstruction were determined. If primary postnatal treatment of cLUTO was carried out in our pediatric urology department, we included these patients for further analysis (Figure 1). Patients' characteristics (sex, gestational age and birth weight) as well as the underlying pathology of cLUTO were assessed. Furthermore, the type and time point of initial postnatal management (diversion by a urinary catheter, vesicostomy or through VAS) and definite treatment (endoscopic valve incision or vesicostomy due to other urethral pathologies) were evaluated. Demographic factors were described using median and range for continuous variables, as well as absolute and relative percentage frequencies for categorical variables.

### General Indication for Intrauterine Intervention

Indications for antenatal VAS placement were bilateral severe hydronephrosis associated with a thick-walled megacystis and oligo- or anhydramnios. Amniotic fluid plays an essential role in fetal lung development and its absence may lead to pulmonary hypoplasia causing a life-threatening condition. According to recent findings, early fetal intervention can be considered at the end of the first trimester, if the following applies: (1) a longitudinal bladder diameter > 12 mm, (2) a normal nuchal translucency (as a screening to detect chromosomal abnormalities), and (3) umbilical cord cysts could be ruled out (as a hint for urethral atresia) (10).

### Treatment Algorithm

**Figure 2:**
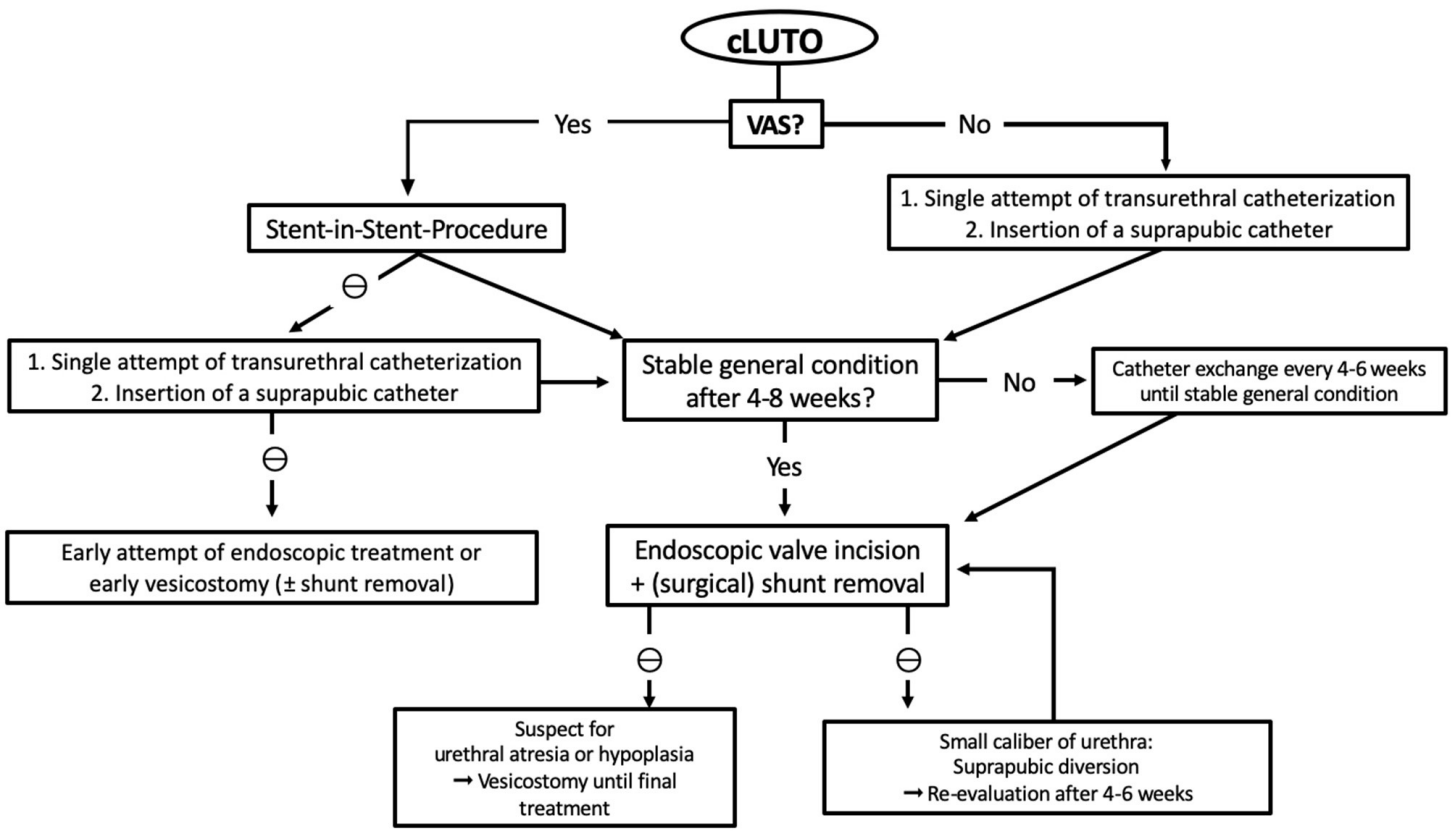
Flowchart presenting the therapy algorithm of our institution for the postnatal management of patient suspected for cLUTO. cLUTO: congenital urinary tract obstruction, VAS: vesicoamniotic shunt, ⊖: if not possible.

In our center, we follow an algorithm for postnatal management of cLUTO patients, as shown in Figure 2. The treatment depends on whether a vesicoamniotic shunt is in place or not.

In newborns *without* VAS, we once attempt transurethral catheterization for urinary drainage directly after birth. Routinely, we use 3–5 Fr feeding tubes or 6 (-8) Fr silicone foley catheters (without mandrins). If transurethral catheterization is not successful, suprapubic drainage is preferred until further or definite treatment. We use the pediatric punction set (Cystofix®) with a 10 Fr catheter and a curled pigtail tip.

If a VAS is in place, we first recommend a stent-in-stent drainage. Should this procedure prove unsuccessful, we proceed as mentioned above and place a transurethral or suprapubic catheter.

The further, or even definite, therapy is determined by the newborns' general constitution and co-existing medical conditions or anomalies. If the newborn is clinically stable, an (early) urethrocystoscopy with a 6 Fr cystoscope can be performed at 4–8 weeks of age. Our first-line procedure is the endoscopic valve incision using an 8 Fr resectoscope with a hook-shaped cold knife. We do not use bugbee electrodes or laser fibers for urethral valve ablation to avoid strictures.

## Results

### Patient Characteristics'

Between 2016 and 2020, a total of 209 patients with an anomaly of the lower urinary tract were treated at our tertiary care center. After exclusion of the diagnoses meatal stenosis, exstrophy-epispadias complex and urachal anomalies, 71 patients with a congenital subvesical obstruction remained. There were 31 patients who received primary postnatal treatment of cLUTO in our pediatric urology department, all of which were included for further analysis.

A single patient was female and the remaining 30 patients were male. Prenatal VAS shunting was performed in 17 neonates (55%), while 14 (45%) had no prenatal intervention due to the diagnosis being unknown or the parents' preference for a conservative management (Figure 1).

The median week of gestation at delivery was 36^+2^ (range: 29^+2^-40^+1^ weeks) in the VAS-group, including seven preterm neonates. On the contrary, infants *without* VAS were born at a median gestational age of 38^+6^ weeks (range: 32^+2^ - 41^+5^ weeks). Neonates *with* VAS had a lower median birth weight (2,450 g, range: 1,540–3,390 g) compared to the group *without* VAS (3,088 g, range: 2,710–4,160 g).

### Postnatal Diagnosis

Within the 31 infants who were postnatally managed in our center, the final diagnosis was predominantly posterior urethral valves (*N* = 24, 77%), either isolated (*N* = 21, 68%) or combined with a urethral hypoplasia (*N* = 3, 10%). Other diagnoses included urethral atresia (*N* = 1, 3%), Prune belly syndrome (*N* = 2, 6%) or an undefined anomaly (*N* = 1, 3%). Three neonatal deaths occurred within seven days after birth (10%). Postmortem, the parents rejected an autopsy; hence the underlying pathology remains unkown.

In the group *with* VAS, 8/17 infants were diagnosed with isolated posterior urethral valves and 3/17 in combination with a urethral hypoplasia. Further underlying anomalies included urethral atresia (*N* = 1), Prune belly syndrome (*N* = 1) and an undefined complex urogenital-anorectal malformation in a female (*N* = 1). Furthermore, in three cases the final diagnosis remained unknown due to postnatal death. Among the 14 infants *without* VAS, 13 had isolated PUV and one had a Prune belly syndrome.

### Postnatal Management

Whether a newborn has a VAS *in situ* or not, determines the postnatal treatment by the pediatric urologist. Accordingly, we compared the primary management after birth between the following two groups: cLUTO *without* VAS and cLUTO *with* VAS.

**Figure 3:**
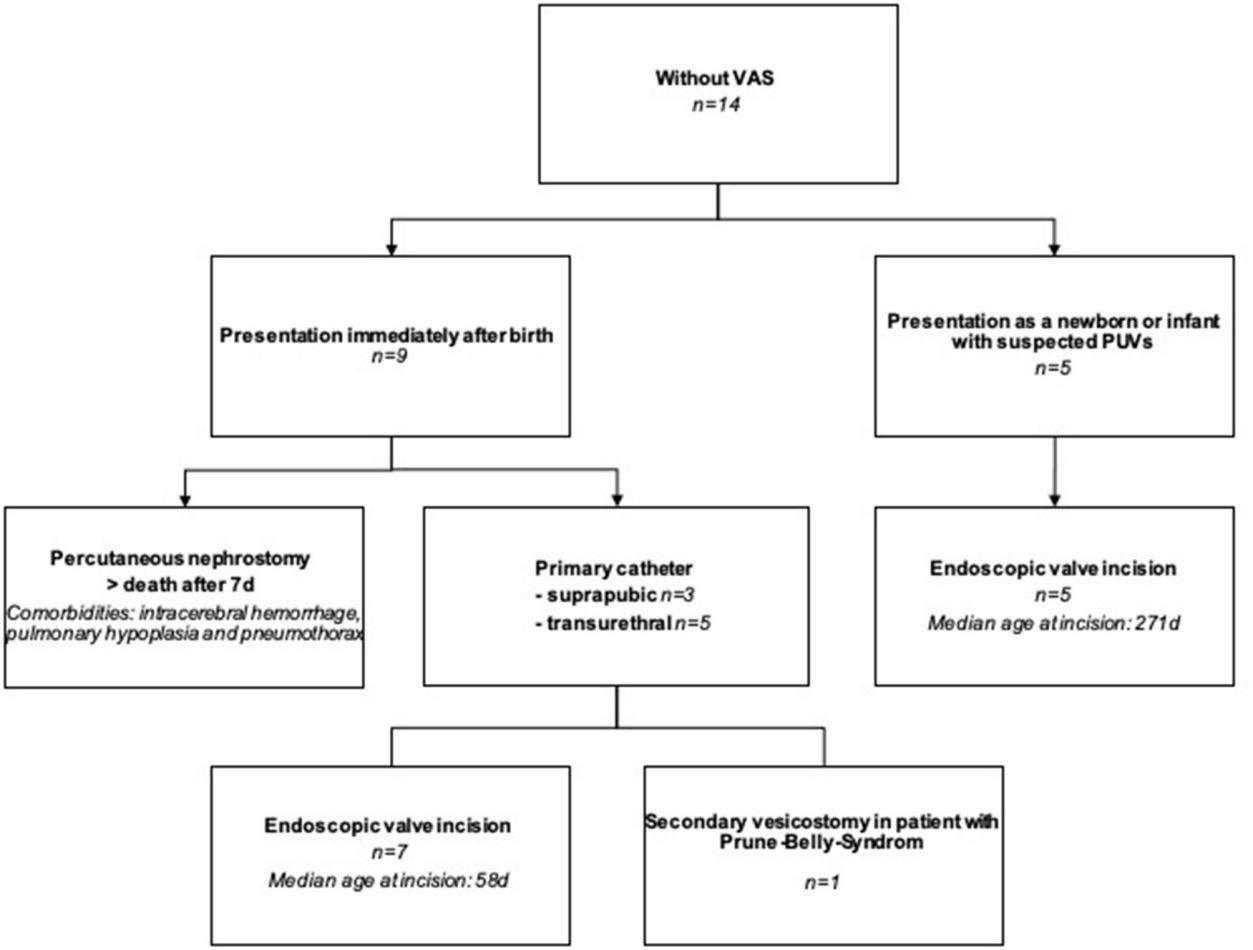
Flowchart showing the treatment of cLUTO patients without VAS after birth. VAS: vesicoamniotic shunting.

Patients *without* VAS were presented to our department either immediately after birth (*N* = 9) or later (*N* = 5). The following procedures were performed on the 14 cLUTO patients *without* VAS (Figure 3):

(1) In seven cases, a urinary catheter was placed until definite urethral valve incision: Two had a suprapubic catheter, another two had a transurethral tube and in the remaining three patients, the initial transurethral drainage was later changed to a suprapubic diversion.
(2) Early vesicostomy at an age of 17 days was performed in one newborn, after initial suprapubic urine drainage.
(3) In a single case, the neonate presented with life-limiting comorbidities (lung hypoplasia, pneumothorax and intracerebral hemorrhage). Due to the obstructive uropathy and anuria, a percutaneous nephrostomy was placed. However, death occurred within the first seven days after birth.
(4) Five infants were presented at a later time point with symptoms suggestive of PUV (e.g., difficulty urinating, weak stream of urine, enlarged bladder with a thick wall in the ultrasound). Here, primary urethral valve incision was carried out without prior urinary drainage.

**Figure 4:**
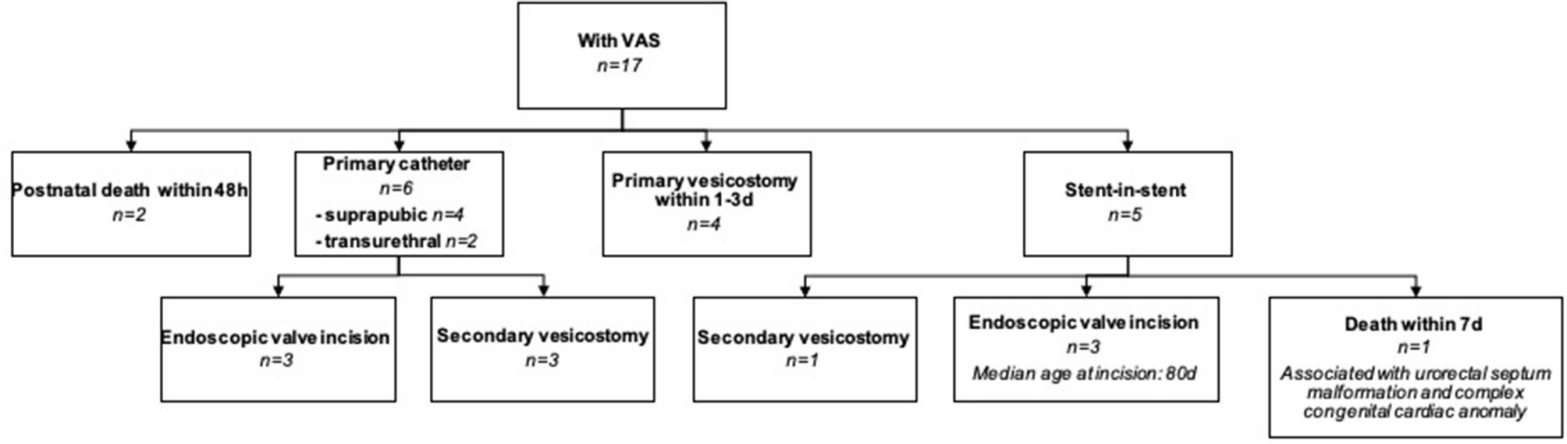
Flowchart explaining the postnatal management of cLUTO newborns with a VAS in place. VAS: vesicoamniotic shunting.

Postnatal management of the 17 infants with cLUTO and a VAS indwelling included (Figure 4):

(1) Initial transurethral (*N* = 2) or suprapubic (*N* = 4) catheterization followed by either a valve incision (*N* = 3) or a secondary vesicostomy (*N* = 3; because a primary urethral valve incision was not possible due to the narrow urethral caliber).
(2) Early vesicostomy within the first 3 days of life (*N* = 4).
(3) Diversion by a tube through the existing VAS, a stent-in-stent-procedure (*N* = 5).
(4) Neonatal death within 48 h (*N* = 2). In one patient a transurethral tube was inserted after birth. The second infant died in the first hour postnatal, hence no tube was inserted.

### Postnatal Urinary Diversion Through VAS

**Figure 5:**
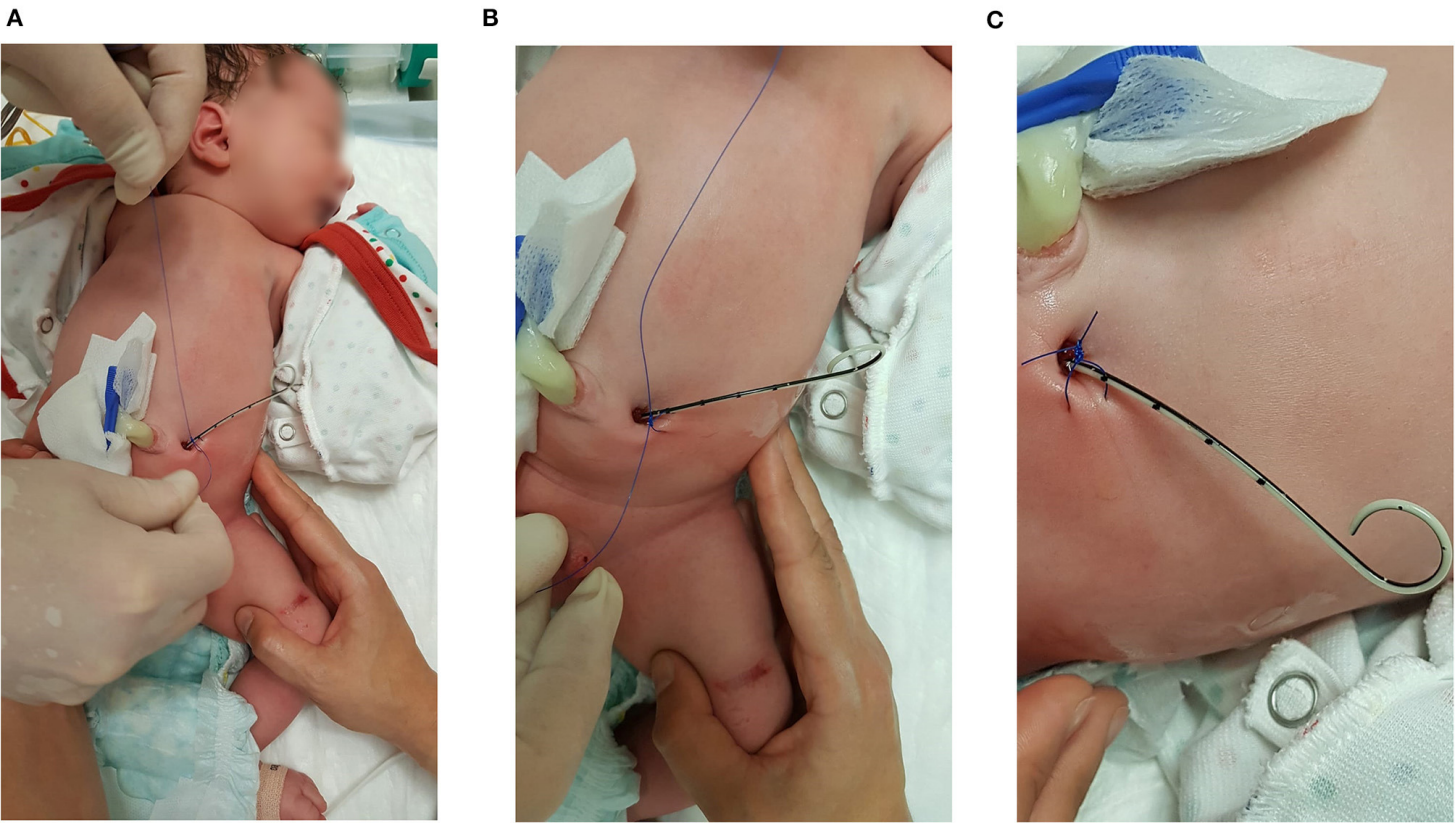
Stent-in-stent scheme with a 4.8 Fr JJ-catheter. The JJ-stent is fixed with a double knot on the skin surface close to the shunt exit site.

In five neonates *with* VAS (Somatex®) we secured urine drainage immediately after birth by placing a gastric tube (*N* = 1) or a 4.8 Fr JJ-stent (*N* = 4) through the lumen of the shunt (Figure 5). There were no patients with a Harrison fetal bladder stent in our cohort.

Within this group, the stent-in-stent diversion was later replaced by a suprapubic catheter in two cases: (1) Further treatment–including the placement of the suprapubic catheter–was continued in an external center. (2) A cystoscopy was performed at an age of 31 days, but an incision was not possible due to the narrow urethral caliber. After 37 days, the urethra was passed endoscopically without signs of hypoplasia and the valves were incised. In two other infants, the JJ-stent remained indwelling until the cystoscopy with definite urethral valve incision (for 55 days and 102 days, respectively). However, in one patient, the urethra was too narrow for intervention after 55 days. As a result, we suspected a urethral hypoplasia and performed a Blocksom vesicostomy. In another patient, urine drainage was secured by a JJ-stent but he died within seven days due to life-limiting comorbidities (urorectal septum malformation and complex congenital cardiac anomaly including a double outlet ventricle).

## Discussion

Up to 1/3 of patients with cLUTO require renal replacement therapy in the long-term (5–7). Since 1983, an increasing number of affected fetuses treated with vesicoamniotic shunting has been reported (9). VAS are typically placed early, between the 12th and the 14th week of gestation. This not only improves the survival rate of fetuses with oligo- or anhydramnios and associated pulmonary hypoplasia (11), but could also enhance the perinatal and the long-term renal function. Interventions performed at advanced stages in pregnancy are mainly beneficial for the fetal lung development, but not for the renal function. The PLUTO-trail – the only prospective, randomized trail analyzing the potential benefits of VAS – showed no difference between the two groups concerning the long-term renal function (12). In 2019, the latest meta-analysis from Chen et al. which included 13 studies analyzing the outcomes and prognostic factors associated with fetal megacystis showed, that intrauterine intervention did not significantly improve prognosis and survival rate (13).

The main challenge of VAS is to identify fetuses which will benefit from antenatal treatment and need intervention. First, there is a strong association between megacystis and chromosomal or structural abnormalities which further impair the prognosis (14). In 90% of the cases with a normal karyotype, megacystis (7–15 mm) resolves spontaneously before the 20th gestational week. On the contrary, spontaneous resolution is unlikely in neonates with a bladder length >12 mm before the 18th week or >15 mm at the 20th week of gestation (15–17). In 2019, Fontanella et al. published a prognostic score to predict the severity of cLUTO using antenatal diagnostics. They stated that gestational age at the occurrence of oligo- or anhydramnios as well as urinary bladder volume at diagnosis can predict morbidity and mortality with a sensitivity of ~78%. Although this scoring system was developed in the Netherlands, it is mainly used in the USA (at the moment).

Since the beginning of the 21^st^ century, the role of fetal urine biochemistry in identifying candidates for in-utero intervention and predicting fetal outcome has been discussed (18). A sodium level <100 mmol/l, a chloride value <90 mmol/l and an osmolarity <200 mOsm/l in three consecutive fetal urine samples gained on three different days seem to indicate a better prognosis (19). Urinary biomarkers such as β-2-microglobline, sodium, chloride and calcium in urine samples before the 23rd week of gestation may be helpful to identify candidates for fetal therapy (20). Buffin-Meyer et al. could demonstrate in their most recent study, that the tested fetal urine peptide signature (12PUV) predicted postnatal renal outcome for suspected PUV with an AUC of 0.89 and an accuracy of 84% (21). Multi-center studies are necessary to confirm these findings.

Currently, European reference networks for rare urological disorders (ERN eUROGEN) and rare kidney diseases (ERN ERKNet) support such a study to generate reliable data (22). The status of amnion fluid, the renal ultrasound as well as the fetal urine biochemistry could be helpful in counseling prospective parents. The desirable aim for the future is to reliably predict, who would benefit from an early fetal intervention.

Nevertheless, after the baby is born pediatric urologists have to tend to the “surgical” management. At the moment, there are several shunts commercially available and more are being developed (23, 24). Some of the VAS, like the Somatec Shunt®, have a larger lumen than the Rocket and Harrison shunt. As most shunts are placed at an early stage of pregnancy, urinary flow is diverted from the fetal urethra. Hypothetically, this could lead to a delayed development of the urethra causing further hypoplasia. In these cases, a transurethral catheterization is not possible. However, this is still a theory based on our own experience without any evidence.

The infants are mostly premature and/or small for gestation, hence they have an increased risk from general anesthesia. In the first 2 years (of our experience in postnatal treatment), we removed the inserted stents in general anesthesia, performed a temporary, incontinent Blocksom vesicostomy (25) and let the neonate grow, until a definitive treatment could be performed. However, performing surgery in this early stage of life can lead to a prolonged duration of ventilation and intensive care unit stay, as some of the newborns suffer from additional severe comorbidities or malformations.

In case of a VAS with a lumen surrounded by nitinol/metal, we placed a gastric tube (3–5 Fr) through the stent into the bladder to secure urine drainage. Since this procedure was successful and acute postnatal surgery could be avoided, we applied the stent-in-stent scheme (4.8 Fr JJ-catheter) in the following four cases. Later, these catheters or stents can be used to perform a VCUG, avoiding any manipulation through the hypoplastic urethra. Securing urine drainage in this manner allows the definitive treatment to be postponed until the early neonatal period is over and the infant is more stable. Moreover, the time gained could be used for more detailed diagnostics and further counseling of the parents.

## Conclusion

Pediatric urologists are challenged with the management of vesicoamniotic stents in cLUTO patients after birth. In our opinion, having a secure urine drainage through the VAS offers the possibility to wait until the mostly premature infants have stabilized and grown. Hereby, the risk of general anesthesia is reduced and the urethral caliber can increase to allow cystoscopy. Hence, transurethral manipulation or a vesicostomy can be avoided.

## Data Availability Statement

The raw data supporting the conclusions of this article will be made available by the authors, without undue reservation.

## Ethics Statement

The studies involving human participants were reviewed and approved by (Ethical Approval No. 2020-873R). Written informed consent to participate in this study was provided by the participants' legal guardian/next of kin.

## Author Contributions

MJ: collection of the data, analysis, wrote part the manuscript, and creation of Figure 1. MJ and NY: collection of the data, analysis, wrote part of the manuscript, and supervision of the results. RS: had the idea, wrote part of the manuscript, and supervision. All authors contributed to the article and approved the submitted version.

## Conflict of Interest

The authors declare that the research was conducted in the absence of any commercial or financial relationships that could be construed as a potential conflict of interest.
